# Deletion of V483 in the spike confers evolutionary advantage on SARS-CoV-2 for human adaptation and host-range expansion after a prolonged pandemic

**DOI:** 10.1038/s41422-024-01000-8

**Published:** 2024-07-19

**Authors:** Can Yue, Shuo Liu, Bo Meng, Kaiyue Fan, Sijie Yang, Pan Liu, Qianhui Zhu, Xin Mao, Yuanling Yu, Fei Shao, Peng Wang, Youchun Wang, Ravindra Kumar Gupta, Yunlong Cao, Xiangxi Wang

**Affiliations:** 1 CAS Key Laboratory of Infection and Immunity, National Laboratory of Macromolecules, Institute of Biophysics, Chinese Academy of Sciences, Beijing, China; 2 Changping Laboratory, Beijing, China; 3 Chinese Academy of Medical Sciences & Peking Union Medical College, Beijing, China; 4 Institute of Therapeutic Immunology & Infectious Disease (CITIID), University of Cambridge, Cambridge, UK; 5 University of Chinese Academy of Sciences, Beijing, China; 6 Biomedical Pioneering Innovation Center (BIOPIC), Peking University, Beijing, China; 7 Joint Graduate Program of Peking-Tsinghua-NIBS, Academy for Advanced Interdisciplinary Studies, Peking University, Beijing, China

**Keywords:** Molecular biology, Cryoelectron microscopy

Dear Editor,

Severe acute respiratory syndrome coronavirus 2 (SARS-CoV-2) has undergone continuous evolution since its initial outbreak in 2019. The emergence of Omicron as the dominant variant occurred abruptly after 2022.^1^ A highly mutated Omicron variant, BA.2.86, has recently been identified and reported in multiple countries. The global prevalence of this variant is gradually increasing, as confirmed by the World Health Organization (WHO), which has categorized it as a variant under monitored (VUM).^2^ The Spike (S) protein of BA.2.86 has achieved more than 30 amino acid changes since its divergence from the parental BA.2 strain. Among these changes, the deletion of residue 483 (∆483) in the receptor binding domain (RBD) region stands out (Fig. 1a; Supplementary information, Fig. S1), as deletions in the S protein typically occur in the N-terminal domain (NTD) region of SARS-CoV-2 variants. Interestingly, the absence of residue 483 has been observed in SARS.^3^ Phylogenic and receptor usage analysis revealed that ∆483 is widespread among sarbecoviruses and is not linked to ACE2 binding^4^ (Fig. 1b). Moreover, the deletion is genetically stable and can be sustained. These findings imply that ∆483 does not hinder the interaction of the virus with ACE2. In fact, it sometimes confers advantage since strains harboring the mutation become new prevalent strains. To assess the impact of the sudden deletion of 483 in SARS-CoV-2 and its potential link to the viral adaptation across different hosts, as well as its role in human pandemic, we conducted a multidimensional analysis covering receptor usage and infection capabilities in different hosts, along with the effects of the deletion on cell–cell fusion, S protein cleavage, and immunogenicity. The findings reported here aid in the understanding of the evolutionary dynamics of the virus.

**Fig. 1:**
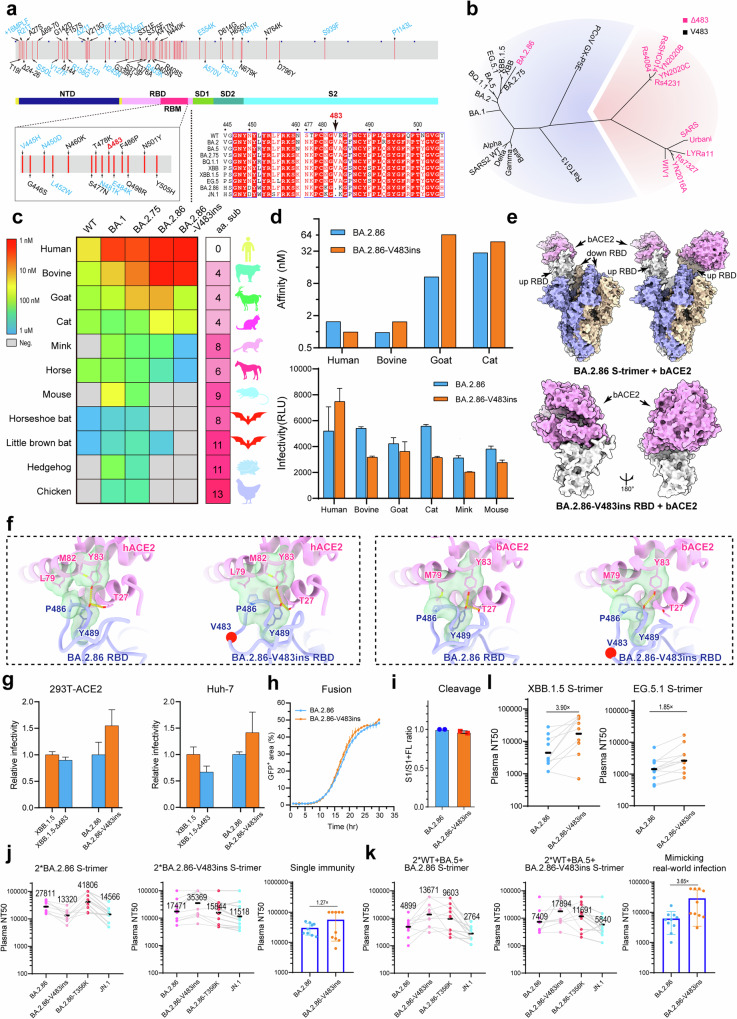
The influence of V483del on SARS-CoV-2 viral fitness. **a** Diagram illustrating the full-length S protein sequence of BA.2.86. Newly added mutations were indicated in blue, while deleted site of RBD was shown in red. **b** Statistical analysis of sarbecoviruses capable of utilizing hACE2. Black font represents coronaviruses retaining residue 483 (WT SARS-CoV-2 sequence number), while red font represents viruses lacking residue 483 (WT SARS-CoV-2 sequence number). **c** Comparison of the affinity of ACE2 from different species for SARS-CoV-2 variants, with species ranked by the amino acid sequence differences from hACE2. **d** Determination of the affinity and infectivity of BA.2.86 and BA.2.86-V483ins to different species. Blue bars represent BA.2.86, and orange bars represent BA.2.86-V483ins. **e** Overall structures of BA.2.86 S-trimer in complex with bACE2 and BA.2.86-V483ins RBD in complex with bACE2. **f** Detailed information on the interactions of hACE2, bACE2 with BA.2.86, BA.2.86-V483ins RBD. Residues involved in the hydrophobic interactions are shown as transparent surfaces and hydrogen bonds are marked with yellow dashed lines. Note that the complex structure of hACE2 and BA.2.86-V483ins RBD was generated using molecular docking. **g** The infectivity of XBB.1.5, XBB.1.5-∆483 and BA.2.86, BA.2.86-V483ins was evaluated in 293T, Huh-7 cells overexpressing hACE2. The evaluation was performed using a *Vesicular stomatitis* virus-based pseudovirus system. Error bars represent the mean ± SD of three replicates. **h** Evaluation of cell fusion ability of BA.2.86 and BA.2.86-V483ins. **i** Evaluation of cleavage efficiency of BA.2.86 and BA.2.86-V483ins. **j**, **k** Single immunity background and mimicking real-world infection background of BALB/c mice were used to evaluate the immunogenicity of BA.2.86 and BA.2.86-V483ins. Left panel, neutralization titers against BA.2.86-derived variants after immunization with BA.2.86. Middle panel, neutralization titers against BA.2.86-derived variants after immunization with BA.2.86-V483ins. Right panel, comparison of neutralization titer of BA.2.86 and BA.2.86-V483ins after immunization with autoantigen. **l** The evasion potential of BA.2.86 and BA.2.86-V483ins against antibodies generated by XBB.1.5 and EG.5.1.

BA.2.86 has been demonstrated to display a high affinity for hACE2.^5–8^ The structural conservation of ACE2 in different species not only makes it possible for many sarbecoviruses to use human ACE2 (hACE2) for infection but also increases the chances of cross-species transmission.^9^ To investigate whether BA.2.86 also exhibits high affinity for ACE2 from animals, we tested wild-type (WT), BA.1, BA.2.75, BA.2.86, and BA.2.86-V483ins for their ability to bind ACE2 from animals under identical conditions (Fig. 1c; Supplementary information, Fig. S2). As anticipated, ACE2 from bovine, goat, and cat, which shows high conservation with hACE2, exhibited a greater compatibility for binding with SARS-CoV-2 and its variants. The affinity gradually increased from WT to BA.2.86, aligning with the trends observed for hACE2. Conversely, ACE2s sharing lower conservation with hACE2 showed limited compatibility in binding with SARS-CoV-2 despite weak binding affinities for BA.1 (Fig. 1c). Interestingly, the addition of V483 was more beneficial to the binding of RBD with hACE2, while for animal-derived ACE2, it was found that the affinity with the receptor decreases by more than 1.6-fold after V483 supplementation (Fig. 1d; Supplementary information, Fig. S2). Additionally, we conducted infection experiments using pseudoviruses of BA.2.86 and BA.2.86-V483ins and tested their ability to infect 293T cells overexpressing either human, bovine, goat, cat, mink or mouse ACE2 (Fig. 1d). The results were consistent with the trends observed in binding affinity studies. The addition of V483 increased the infectivity of cells overexpressing hACE2, but decreased the infectivity of cells overexpressing bovine ACE2 (bACE2), goat ACE2, and cat ACE2.

To investigate the structural basis of these phenomena, we focused on bACE2, which has high affinity for BA.2.86 and BA.2.86-V483ins. Cryo-electron microscopy (cryo-EM) was utilized to determine the structures of BA.2.86 S-trimer in complex with bACE2 and BA.2.86-V483ins RBD in complex with bACE2 (Fig. 1e; Supplementary information, Fig. S3 and Table S1). We conducted local refinements on the cryo-EM reconstructions of these complexes at near-atomic resolution. This refinement process aimed to improve the density around the binding interface between the RBD and ACE2 for enabling a reliable analysis of their interaction modes. Structural comparisons indicate that residue 483 on RBD does not directly participate in the interaction with the ACE2 receptor, but regulates the binding indirectly through conformational alteration of a loop (residues 474–490) where it is located (Fig. 1f). In the context of RBD interacting with hACE2, the loop primarily modulates hACE2 interaction through hydrophobic interactions involving key residues such as L79, M82, and Y83 on hACE2, with residues P486 as well as Y489 on RBD. The presence of residue V483 enhances the flexibility of the loop (Supplementary information, Fig. S4), facilitating a more rational contact between hydrophobic residues P486, and Y489 on RBD and their counterparts on hACE2. As a result, the hydrophobic interaction surface area increases. However, deletion of residue V483 reduces the plasticity of the loop, leading to restricted interaction with hACE2 and diminishing the hydrophobic interaction surface area between RBD and hACE2. It is worth noting that in bovine, goat, and cat ACE2, the conversion of residue 82 to a hydrophilic amino acid, Met→Thr, significantly weakens the hydrophobic binding interactions with the loop of RBD (Supplementary information, Fig. S2), thereby greatly reducing the constraints imposed on loop mobility. The absence of residue V483 reduces the flexibility of the loop, localizing its flexible conformation and promoting the formation of a stable structure upon binding to bACE2. However, the insertion of residue V483 enhances its flexibility even further. This directly results in the loss of hydrogen bonding between Y489 on RBD and T21 on bACE2, leading to a significant reduction in the interaction between RBD and bACE2.

To evaluate whether the deletion of V483 could emerge as a prominent characteristic in epidemic strains and its potential advantages for human adaptation, we conducted additional infectivity tests for both XBB.1.5/BA.2.86 and their derivative variants (with or without V483), using the human cell lines 293T-ACE2 and Huh-7 (Fig. 1g). XBB.1.5, XBB.1.5-∆483, BA.2.86 and BA.2.86-V483ins exhibited consistent results in all the cell lines tested, where the strains without V483 displayed lower infectivity. Remarkably, addition of V483 based on BA.2.86 doubled the infectivity. We further determined whether the diminished infectivity of the ∆483 strains is correlated with its cell fusion or S cleavage efficiency. The isolated GFP system was used to monitor fusion events involving BA.2.86 and BA.2.86-V483ins (Fig. 1h). It was observed that there was no discernible alteration in cell–cell fusion between BA.2.86-V483ins and BA.2.86. Similarly, there was also no significant difference in the cleavage ability of S protein (Fig. 1i). Hence, the reduction in infectivity of human cell lines caused by the deletion of 483 is brought about by changes in receptor affinity, but has no obvious correlation with the changes in cell fusion and S protein cleavage abilities.

In our previous study on the roles of the N354 glycosylation in immunogenicity and immune imprinting, two groups of mice with different immunity backgrounds were established: one was immunized by one single immunogen (BA.5, XBB.1.5, EG.5.1, BA.2.86 or BA.2.86-T356K S-trimer), while the other group was sequentially administrated with various immunogens mimicking real-world natural infection (two doses of WT inactivated vaccines, one dose of BA.5 inactivated vaccine, one dose of recombinant BA.5, XBB.1.5, EG.5.1, BA.2.86 or BA.2.86-T356K S-trimer)^10^ (Supplementary information, Fig. S5). Herein, we added the BA.2.86-V483ins-vaccinated mice in each group to evaluate the effect of 483 on immunogenicity and immune imprinting. From the analysis of a single immune background, although both BA.2.86 and BA.2.86-V483ins can induce high levels of neutralizing titers against the BA.2.86 sublineages, a notable antigenic drift was clearly observed with 1.6–2.7-fold (27811/17471, 35369/13320) neutralization differences raised by the residue 483 (Fig. 1j; Supplementary information, Fig. S6). Furthermore, BA.2.86 demonstrated ~30% lower immunogenicity compared to BA.2.86-V483ins. Distinct with single immunity background, BA.2.86-V483ins elicited a significant improvement in neutralizing titers against all tested SARS-CoV-2 variants when compared to BA.2.86 in mice with a hybrid immunity background (Fig. 1k; Supplementary information, Fig. S6). More importantly, ∆483 resulted in an ~3.65-fold reduction in the immunogenicity under hybrid immunity background. These findings indicated that ∆483 facilitated less cross-reactive memory B cell recall responses, probably benefiting from immune escape and alleviating immune imprinting in current real-world context. To further verify this, we conducted neutralization assays to evaluate the antibody evasion of BA.2.86 and BA.2.86-V483ins against sera from hybrid immunization; a mimicry of real-world infections prior to BA.2.86 emergence (Fig. 1l; Supplementary information, Fig. S7). In comparison with BA.2.86-V483ins, ∆483 conferred ~1.2–4-fold stronger immune evasion under mimicry of BA.5, BA.2.75, XBB.1.5, EG.5.1 breakthrough infection background, explaining the prevalence of BA.2.86 sublineages. These observations suggest a viral evolution trade-off by compromising infectivity in exchanging for an expanded host range, greater evasion of immunity and lower immune imprinting for a higher goal of co-existence with humans.

Our structural and functional analysis reveals the following potential implications. 1) The absence of V483 leads to varying receptor affinity and infectivity of BA.2.86 across different species, highlighting potential species-specific adaptability. 2) The deletion of V483 may confer a higher risk of animal infection for BA.2.86 relative to humans. 3) The loss of V483 results in reduced immunogenicity and immune imprinting of BA.2.86, while enhancing its evasion capability, indicating that the virus confers a strong advantage for reinfections by future variants. 4) Regarding the development of an updated (BA.2.86-based) vaccine, the V483 supplementation can serve as an optimal strategy to elicit improved neutralizing titers against BA.2.86 sublineages. In summary, our study provides insights into the evolution of the BA.2.86, offering valuable understanding of virus–host interactions and significant implications for the development of therapeutic strategies targeting emerging SARS-CoV-2 variants. However, this study is subject to certain limitations. Due to the lack of access to blood samples from individuals infected with or vaccinated against the BA.2.86-V483ins variant under natural conditions, mice were utilized as model organisms to assess the immunogenicity and neutralization capabilities of both the BA.2.86 and BA.2.86-V483ins variants. Therefore, there may be slight differences when applying these findings to humans.

## Supplementary information

Supplementary Information

## Data Availability

The atomic coordinates of BA.2.86 S trimer with 1 bACE2, BA.2.86 S trimer with 2 bACE2, BA.2.86 S trimer with 1 bACE2 of local refinement and BA.2.86-V483ins with bACE2 have been deposited in the protein data bank (PDB) under accession codes 8XZ8, 8XZ9, 8XZA, 8Y32, respectively. Cryo-EM density maps for these complexes have been deposited at the Electron Microscopy Data Bank (EMDB) with accession codes EMD-38789, EMD-38790, EMD-38791, EMD-38866, respectively. It is worth noting that the structural data of hACE2 and BA.2.86 refer to the previously reported structure (PDB 8WHZ). The complex data of hACE2 and BA.2.86-V483ins adopt those of hACE2 (PDB 8WHZ) docking with BA.2.86-V483ins S-trimer (PDB 8X4Z).
