# Supplementary material for: Deletion of V483 in the spike confers evolutionary advantage on SARS-CoV-2 for human adaptation and host-range expansion after a prolonged pandemic
Source: Cell Res. 2024 Jul 19;34(10):739–42. doi: 10.1038/s41422-024-01000-8 (PMC11442493; doi:10.1038/s41422-024-01000-8)
Supplement: Supplementary file 1 — Supplementary Information [file 41422_2024_1000_MOESM1_ESM.pdf]

Supplementary information

Method

Protein expression and purification

The spike (S) and RBD genes of BA.2.86 (GenBank:WMV03218.1) were obtained by overlapping PCR, using the BA.2.75 gene as a template. The BA.2.86-V483ins S and RBD genes were obtained by introducing mutations to the BA.2.86 S and RBD genes. Animal-derived ACE2 genes (**Table S2**) were cloned into PCCAGGS vector for protein expression (synthesized by Genescript). To stabilize the conformation of S- trimer, proline substitution was performed on residues 817, 892, 899, 942, 986 and 987 in all Spike gene constructions, and 2A mutation was introduced at R683A and R685A positions. Furthermore, a T4 fibritin folding domain was added to the end of the sequence for modification. To facilitate protein purification, the C-terminal of all gene constructs was tagged with 2×Strep II and 6×His. The protein was produced using the eukaryotic system. Plasmid containing the S or RBD gene was transiently transfected into HEK293F cells and cultured in a constant-temperature shaker with 8% CO_2 at 37 °C for over 72 hours. After the incubation time, the cell supernatant was collected, followed by preliminary protein purification using Ni-NTA or affinity StrepTactin resin chromatography. To achieve high purity, the RBD was re-purified with Superdex 200 10/300GL (Cytiva) in phosphate-buffered saline (PBS) at a pH of 7.4. Similarly, the S-trimer was re-purified using a Superose 6 10/300 (Cytiva) column.

Surface Plasmon Resonance

The affinity between the antigen and receptor was determined using Surface Plasmon Resonance (SPR) technology. ACE2 from different sources were immobilized on a CM5 chip (Cytiva) and used as the stationary phase, while BA.2.86 RBD and 2.86-V483ins RBD were used as the mobile phase. The experiments were conducted at a temperature of 25°C using a Biacore 8K biosensor for data detection and recording. The raw data were assessed and fitted using the evaluation software of Biacore 8K (GE Healthcare) with a 1:1 binding model.

Cryo-EM sample preparation

The purified protein sample was centrifuged at high speed to remove the precipitate. The final concentration of the sample was adjusted to 1.0 mg/mL. To prepare the S-trimer/hACE2 complex sample, S-trimer and ACE2 were mixed at a molar ratio of 1:3.6. Porous carbon-coated gold grids (C-flat, 300 mesh, 1.2/1.3, Protochips Inc.) were glow treated under H_2, O_2 conditions for 21-25 seconds. The Vitrobot (FEI), a punch-in freezing equipment, was used in the powerless mode at 100% relative humidity and 22°C. 3 μL of the sample was placed on the grid and the sample was then blotted dry for 6 seconds. The sample was quickly immersed in liquid ethane and frozen.

Data collection and model building

The samples were collected using a 300 kV FEI Titan microscope (Thermo Fisher) equipped with K2 and K3 detectors. The SerialEM software was used to automatically collect the data. The collection parameters included a total electron dose of 60 e–$Å^{-2}$, 32 frames with an exposure time of 0.2 seconds per frame, and a pixel size of either 1.04 Å or 1.07 Å. CryoSPARC (version 4.3.0) or Relion (version 3.0.8) were employed to determine the data obtained through cryo-electron microscopy. The processing steps included motion correction, CTF estimation, template creation, template selection, photomicrograph extraction, 2D classification, 2D selection, *Ab-initio* reconstruction, and homogeneous refinement. For regions requiring higher resolution, such as the RBD/RBD-ACE2 region, local refinement was conducted using UCSF Chimera (version 1.13.1) and CryoSPARC (version 3.2.1). The structure was modeled using WinCoot (version 0.9.8.1), while the refinement and verification of the structure were carried out with Phenix (version 1.20.1). The figure was generated using UCSF ChimeraX (version 1.7.0).

MD simulation and RMSF calculating

Initial models of BA.2.86 and BA.2.86-V483ins RBD were from the structure

determined by this study. CHARMM-GUI was used to generate the inputs for simulation packages GROMACS before final simulation. Briefly, after PDB checking, waterbox size specifying, water model specifying (TIP3P), ions adding, periodic boundary condition setting and force fielding specifying (OPLS-AA/M) steps, the data generated were submitted to GROMACS-2022. After Energy Minimization, NVT Equilibration, NPT Equilibration, 5 ns MD simulation was carried out. NVT ensemb1e via the Nose-Hoover method at 300 K and NPT ensemble at 1 bar with the Parinello-Rahman algorithm were employed to make the temperature and the pressure equilibrated, respectively. The frames for last 2 ns were extracted to calculated RMSF. Figures were generated in ChimeraX (version 1.7.0).

Pseudovirus infectivity assay

The S protein gene were optimized for mammalian codons and inserted into the pcDNA3.1 vector. Plasmids were transfected into 293T cells using Lipofectamine 3000 (Invitrogen). These cells were then infected with G* Δ G-VSV pseudovirus (Kerafast) and SC2-VLP pseudovirus, respectively. After incubation, the pseudovirus was collected and filtered using a 0.45 μm filter membrane. The virus was then sub-packed and stored at -80 °C.

HEK293T-ACE2 cells (human, bovine, sheep, cat, mink, mouse) were used as the target for detecting pseudovirus infection. After quantification using RT-PCR, the diluted virus was introduced into a 96-well cell culture plate and incubated in a 5% CO_2 37 °C incubator for 20-24 hours. A mixture of luciferase substrate and cell lysis buffer (PerkinElmer, Fremont, CA) was added to the plate and incubated in the dark for 2 minutes. Then, the cell lysate was transferred to another plate for detection. The luminescent value was measured using a micro-orifice spectrophotometer (PerkinElmer, HH3400). The IC50 value was determined by fitting the logistic regression model.

Cell-cell fusion assay

HEK293T GFP11 and Vero-GFP1-10 cells were utilized for cell fusion experiments.

When the cells reached 80% density, the two types of cells were seeded into the culture plate at a 1:1 ratio. The cells were then transfected with 0.5 μg of BA.2.86/BA.2.86-V483ins plasmid using Fugene 6 (Promega) transfection reagent. Subsequently, Incucyte was employed to observe and record the fusion of cells, and the ratio of the green area to the total area was calculated.

Cleavage assay

Cleavage experiments for S protein were as described previously[10]. Briefly, cells were washed and lysed using lysis buffer (Cell Signalling). The lysate was then diluted with 4× sample buffer (Bio-Rad) and boiled for 10-15 minutes. Subsequently, mouse anti-SARS-CoV-2 S1 antibody (MAB105403, R&D Systems), rabbit anti-SARS-CoV-2 S monoclonal antibody (PA1-41165, Thermo Fisher Scientific), and horseradish peroxidase (HRP)-conjugated anti-rabbit and anti-mouse IgG polyclonal antibody (Cell Signalling) were used for Western blot analysis. The cleavage ratio of S1 to full-length (FL) in virions was quantified using ImageJ software (NIH).

Assessment of immunogenicity

To assess the immunogenicity of the BA.2.86/BA.2.86-V483ins variant, two groups of BALB/c mice were established, as previously described[10.] The first group received an intramuscular injection of 10 μg of BA.2.86/BA.2.86-V483ins S-trimer protein (as the antigen) and a booster immunization after 14 days. Blood samples were collected on the 14th day following the second immunization. The second group of mice simulated natural infection by receiving a primary dose of 0.3 μg of CoronaVac (an inactivated vaccine against SARS-CoV-2 WT) at 6 weeks of age, followed by a booster dose 21 days later to simulate WT break through infection (BTI). After 3.5 months, these mice received an additional 0.3 μg of Omicron (BA.5) inactivated vaccine (Sinovac) to simulate BA.5 BTI. Furthermore, after 4 months, they were vaccinated with 10 μg of BA.2.86/BA.2.86-V483ins S protein to simulate BTI + reinfection, and blood was collected on day 14 after immunization.

Supplementary Figures

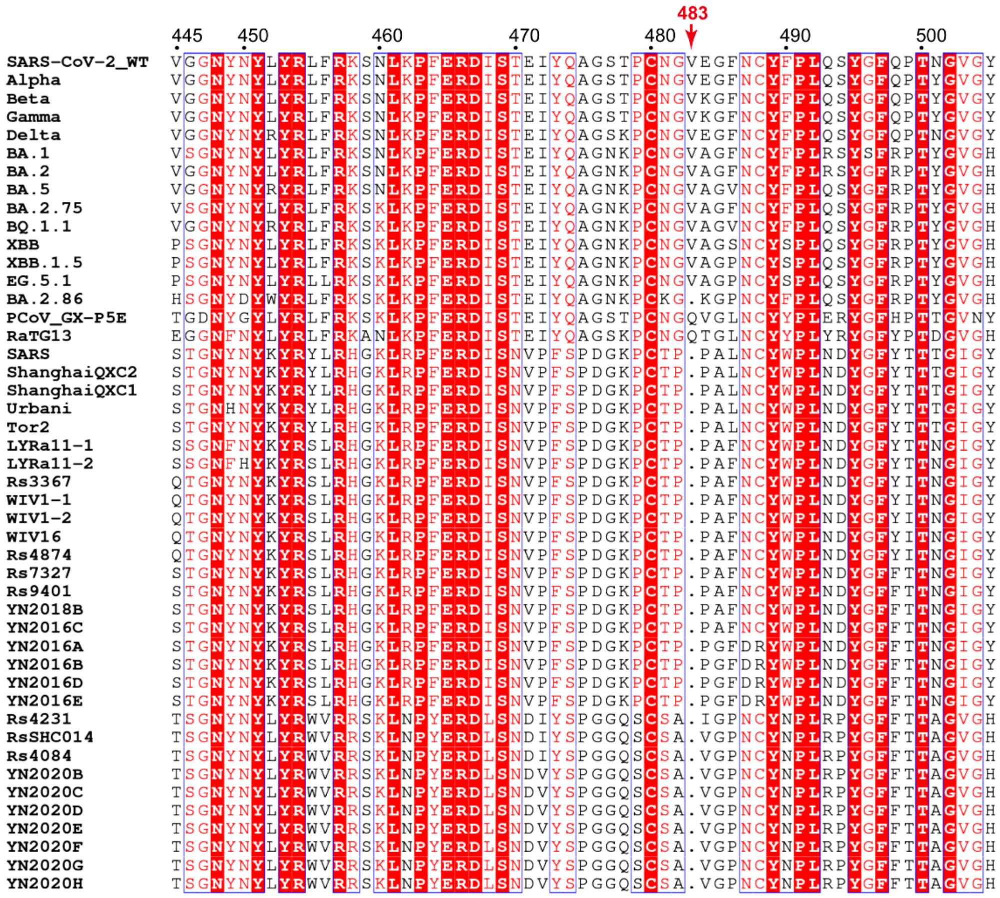

Supplementary information, Fig. S1 Multiple Sequence Alignment of Receptor Binding Motif (RBM) of sarbecovirus strains.

Multiple sequence alignment of the selected sarbecovirus was performed using the sequence of SARS-CoV-2 WT Spike protein as the standard. The position of 483 was marked.

	WT	BA.1	BA.2.75	BA.2.86	BA.2.86-V483	19	24	27	28	30	31	34	35	37	38	41	42	45	79	82	83	325	329	330	353	354	355	357	393
human	19.6	2.47	2.95	1.55	0.99	S	Q	T	F	D	K	H	E	E	D	Y	Q	L	L	M	Y	Q	E	N	K	G	D	R	R
bovine	59.5	14.9	5.34	0.97	1.54	S	Q	T	F	E	K	H	E	E	D	Y	Q	L	M	T	Y	Q	D	N	K	G	D	R	R
goat	86.2	64.1	12.8	10.6	64.8	S	Q	T	F	E	K	H	E	E	D	Y	Q	L	M	T	Y	Q	N	N	K	G	D	R	R
cat	99.1	145	120	29.8	48.1	S	L	T	F	E	K	H	E	E	E	Y	Q	L	L	T	Y	Q	E	N	K	G	D	R	R
mink	neg.	107	159	167	4420	S	L	T	F	E	K	Y	E	E	E	Y	Q	L	H	T	Y	E	Q	N	K	R	D	R	R
horse	108	117	156	358	15300	S	L	T	F	E	K	S	E	E	E	H	Q	L	L	T	Y	Q	E	N	K	G	D	R	R
mouse	neg.	32.6	142	neg.	neg.	S	N	T	F	N	N	Q	E	E	D	Y	Q	L	T	S	F	Q	A	N	H	G	D	R	R
Horseshoe bat	3940	627	526	neg.	neg.	S	E	M	F	D	K	T	K	E	D	H	Q	L	L	N	Y	E	N	N	K	G	D	R	R
Little brown bat	3890	466	327	722	neg.	S	K	I	F	E	N	S	K	E	D	H	E	L	L	T	Y	P	N	N	K	G	D	R	R
Hedgehog	neg.	125	315	neg.	neg.	S	Q	S	F	T	T	N	E	E	N	Y	Q	L	L	K	F	Q	D	K	L	N	D	R	R
chicken	neg.	302	262	neg.	neg.	D	E	T	F	A	E	V	R	E	D	Y	E	L	N	R	F	E	T	N	K	N	D	R	R

Supplementary information, Fig. S2 K_D values of SPR assay and residues on ACE2 from different species that interact with SARS-CoV-2 RBD.

The unit of KD values is nM. Residues the same with and different from human ACE2 are shadowed by blue and pink squares (Note: Some of the data comes from previous study[10]).

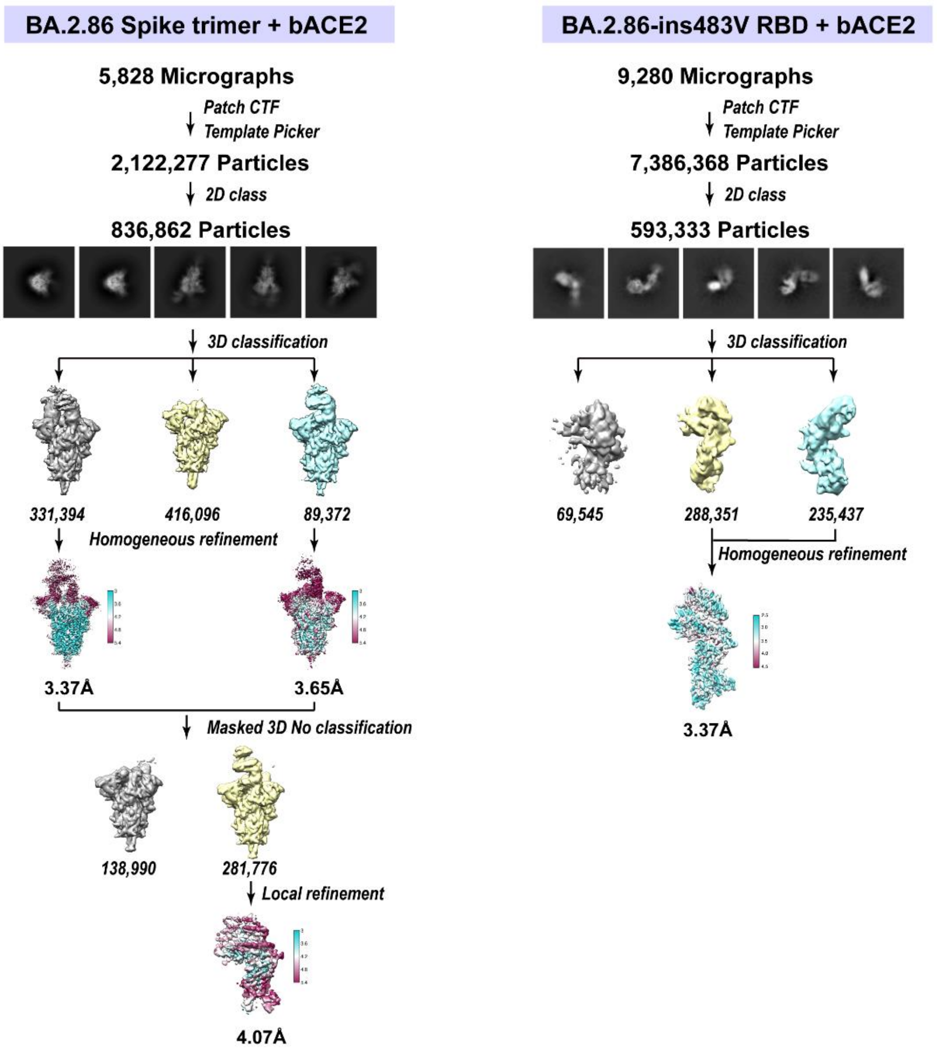

Supplementary information, Fig. S3 Cryo-EM structure of BA.2.86 S-trimer and BA.2.86-V483ins RBD bound to bovine ACE2.

Flowcharts and Resmap for BA.2.86 S-trimer in complex with bovine ACE2 and BA.2.86-V483ins RBD in complex with bovine ACE2.

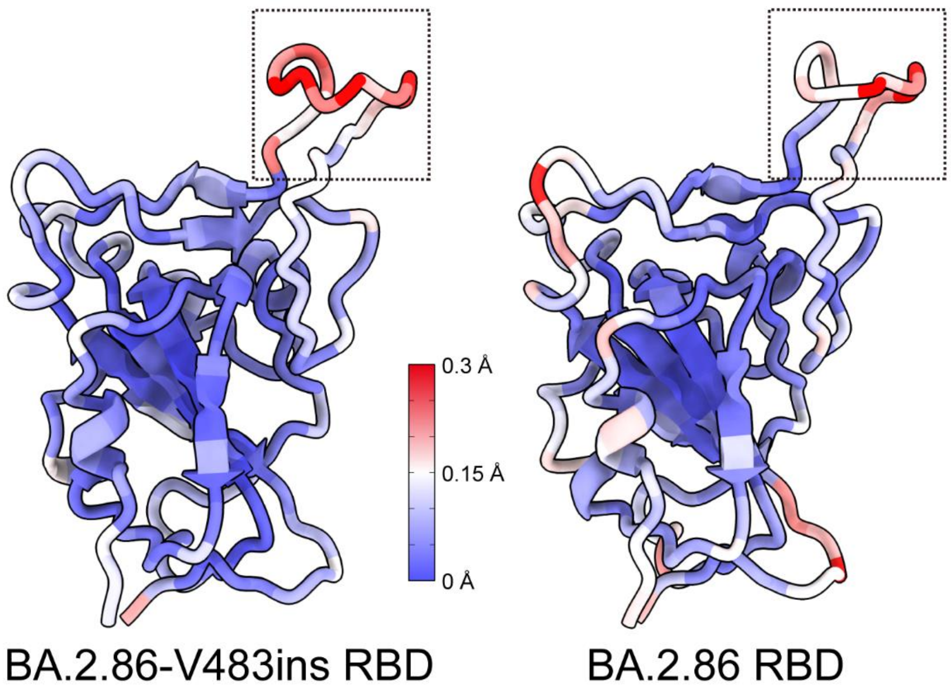

Supplementary information, Fig. S4 The stability landscapes of BA.2.86 and BA.2.86-V483ins RBD.

Molecular dynamics simulations reveal stability alterations in BA.2.86 due to the presence or absence of V483. The cartoons of RBD are colored by root mean square fluctuation (RMSF) calculated from the last 2 ns of the MD simulations. Loops where 483 is located are circled.

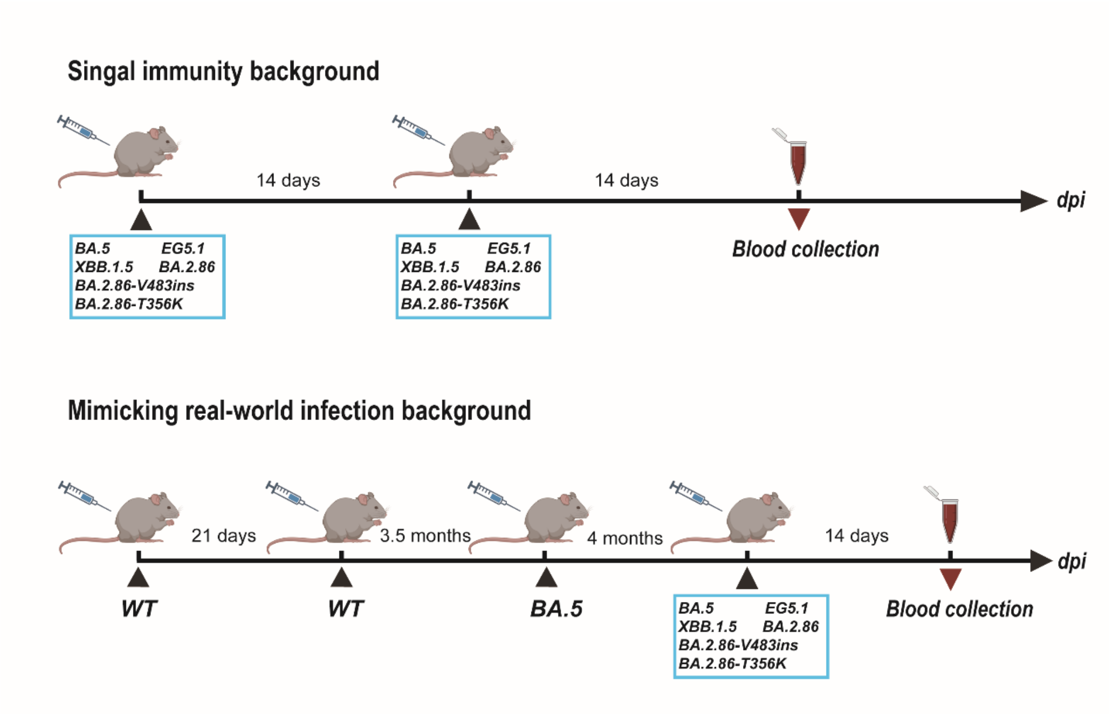

Supplementary information, Fig. S5 The schematic of BA.2.86-V483ins immunization in single immunity background and mimicking real-world infection background.

In the previous study[10] , two groups of mice with distinct immune backgrounds were established: one group received two doses of S-trimer protein immunization using a single immunogen (BA.5 or XBB.1.5 or EG.5.1 or BA.2.86 or BA.2.86-T356K), administered 14 days apart consecutively; the other group was sequentially administered various immunogens that simulated real-world natural infections (two doses of WT inactivated vaccine, one dose of BA.5 inactivated vaccine, one dose of recombinant BA.5 or XBB.1.5 or EG.5.1 or BA.2.86 or BA.286-T356K). Blood samples were collected 14 days after the last immunization. BA.2.86-V483ins recombination was added in the above two groups of mice, respectively.

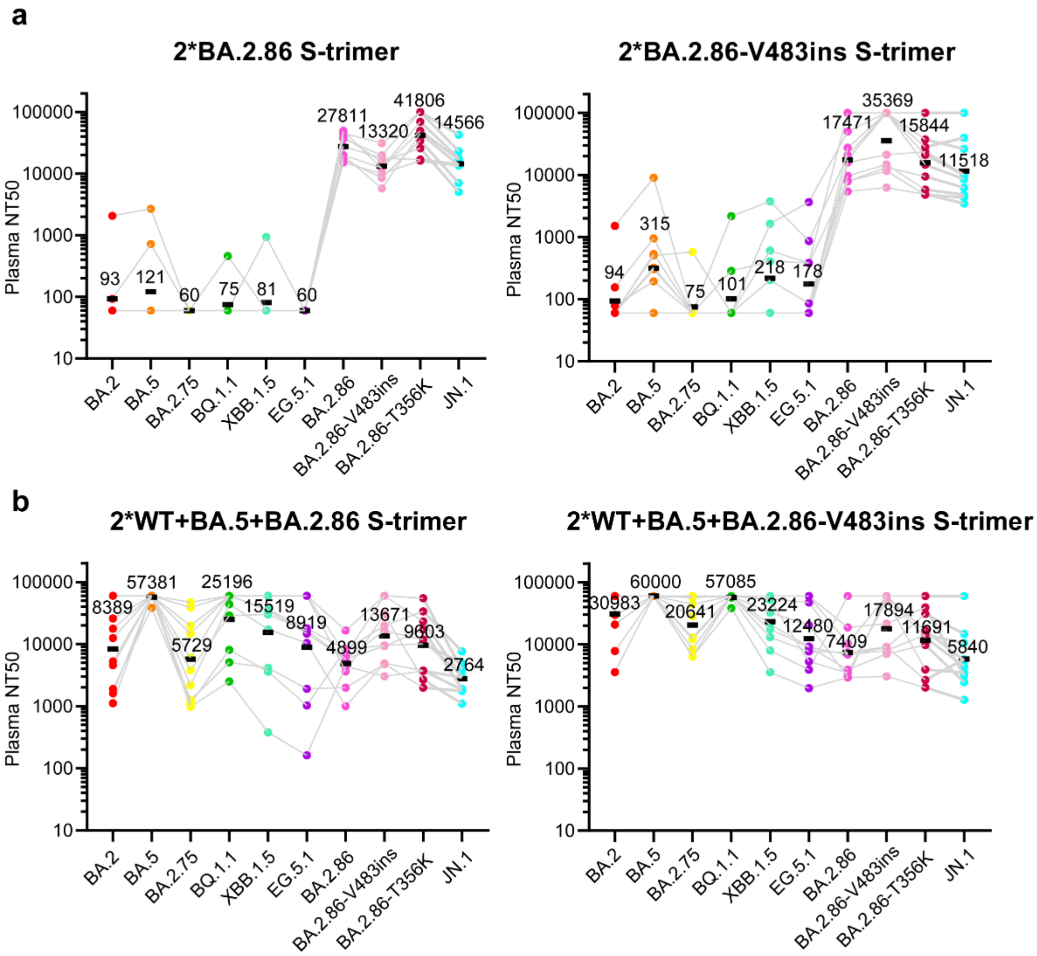

Supplementary information, Fig. S6 483 deletion reduces immunogenicity in hybrid immunity background.

The 50% neutralizing titers (NT50s) against Omicron variants (BA.2, BA.5, BA.2.75, BQ.1.1, XBB.1.5, EG.5.1, BA.2.86, BA.2.86-V483ins, BA.2.86-T356K, and JN.1) in plasma samples from BALB/c mice under a single immunity background (a) and under a simulated real-world infection background (b) are assessed.

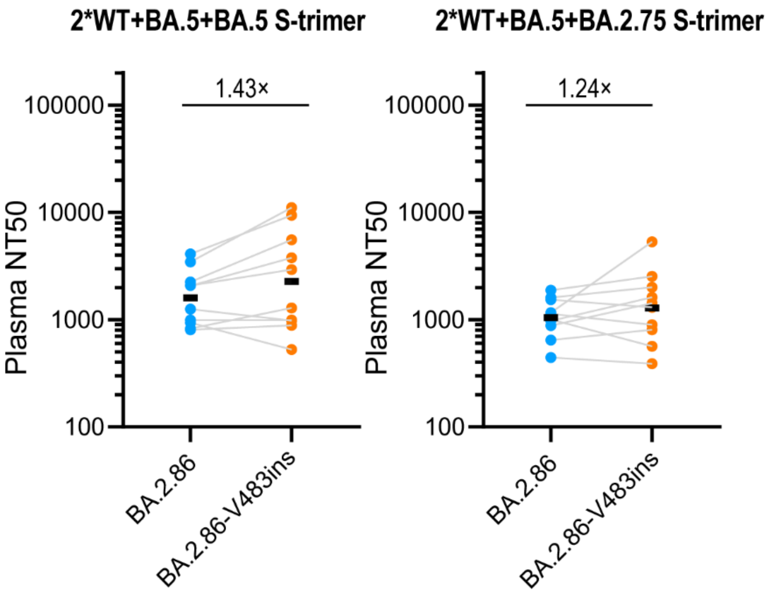

Supplementary information, Fig. S7 483 deletion strengthens ability to escape plasma from BALB/c mice simulating a real-world immune background.

The 50% neutralizing titers (NT_{50}) against BA.2.86 and BA.2.86-V483ins in plasma from mice simulating a real-world immune background are shown.

Supplementary information, Table S1 | Statistics for cryo-EM data collection, refinement, and validation

**Data collection**				
Complex	BA.2.86 S+bACE2 (bound to 1 ACE2)	BA.2.86 S+bACE2 (bound to 2 ACE2)	BA.2.86 S+bACE2 (local refinement)	BA.2.86-V483ins RBD+bACE2
Microscope	FEI Talos Arctica	FEI Talos Arctica	FEI Talos Arctica	FEI Titan
Camera	Gatan K2	Gatan K2	Gatan K2	Gatan K3
Voltage (kV)	200	200	200	300
Total dose (e^-/A^2)	60	60	60	60
Micrographs (total)	5,828	5,828	5,828	9,280
Micrographs (used)	5,828	5,828	5,828	9,280
Particles selected	836,862	836,862	836,862	7,386,368
Particles included in final reconstruction	89,372	331,394	281,776	593,333
sampling, Å per pixel	1	1	1	1.07
Defocus range (μm)	-1.2 ~ -2.0	-1.2 ~ -2.0	-1.2 ~ -2.0	-1.2 ~ -2.0
Symmetry	C1	C1	C1	C1
Resolution (Å) (FSC=0.143 criterion)	3.65	3.37	4.07	3.37
**Model refinement**				
Ramachandran statistics (%)				
Most favored	92.38	92.4	93.13	94.03
Allowed	7.38	7.37	6.74	5.72
Outliers	0.24	0.23	0.13	0.25
Bonds (RMSD)				
Bond lengths (Å)	0.005	0.008	0.004	0.003
Bond angles (°)	0.81	1.006	0.886	0.579
MolProbity score	2.23	2.29	2.31	1.90
Clash score	17.99	20.88	23.86	9.34
Rama-Z				
Rotamer outliers (%)	0.45	0.58	0	0.86
Cβ outliers (%)	0	0.05	0	0
**Deposit**				
PDB	8XZ8	8XZ9	8XZA	8Y32
EMDB	EMD-38789	EMD-38790	EMD-38791	EMD-38866

Supplementary information, Table S2 | Information of species and ACE2 sources

Order	Family	Common name of species	Latin name of species	NCBI number
Bats	Vespertilionidae	Little brown bat	*Myotis lucifugus*	XP_023609437
	Rhinolophidae	Horseshoe bat	*Rhinolophus sinicus*	AGZ48803.1
Carnivores	Felidae	Cat	*Felis catus*	MT663959.1
	Mustelidae	Mink	*Neovison vison*	MT560518.1
Even-toed ungulates	Bovidae	Goat	*Capra hircus*	XM_005701072.3
		Bovine	*Bos taurus*	XM_005228429.4
Odd-toed ungulates	Equidae	Horse	*Equus caballus*	XM_001490191.5
Placentals	Tenrecidae	Hedgehog	*Echinops telfairi*	XP_004710002
Primates	Hominidae	Human	*Homo species*	AB591025.1
Rodents	Muridae	Mouse	*Mus musculus*	NP_081562
Birds	Phasianidae	Chicken	*Gallus gallus*	XP_416822
